# Ending Race-Conscious College Admissions and Its Potential Impact on the Infectious Disease Workforce

**DOI:** 10.1093/ofid/ofae083

**Published:** 2024-02-14

**Authors:** Florence Momplaisir, Tanya Rogo, Ronika Alexander Parrish, Shirley Delair, Mona Rigaud, Virginia Caine, Judith Absalon, Bonnie Word, Dial Hewlett

**Affiliations:** Division of Infectious Diseases, Department of Medicine, Perelman School of Medicine at the University of Pennsylvania, Philadelphia, Pennsylvania, USA; The Penn Leonard Davis Institute of Health Economics, University of Pennsylvania, Philadelphia, Pennsylvania, USA; Division of Pediatric Infectious Diseases, Department of Pediatrics, Warren Alpert Medical School of Brown University, Providence, Rhode Island, USA; Vaccines & Antivirals Medical and Scientific Affairs, Pfizer Biopharmaceuticals Group, New York, New York, USA; Division of Pediatric Infectious Diseases, Department of Pediatrics, University of Nebraska Medical Center, Omaha, Nebraska, USA; Department of Pediatrics at NYU Grossman School of Medicine, NYU Langone Hospital-Brooklyn, Brooklyn, New York, USA; Division of Infectious Diseases, Department of Medicine, Indiana University School of Medicine, Indianapolis, Indiana, USA; Infectious Diseases & Virology, Development Clinical Sciences, GlaxoSmithKline Pharmaceutical, New York, New York, USA; Houston Travel Medicine Clinic, Houston, Texas, USA; Tuberculosis Services, Westchester Department of Health, Chair IDSA Committee on Diversity Access & Equity, White Plains, New York, USA

**Keywords:** health equity, infectious disease specialty, workforce diversity

## Abstract

On 29 June 2023, the Supreme Court of the United States ruled that race-conscious consideration for college admission is unconstitutional. We discuss the consequences of this ruling on the delivery of equitable care and health system readiness to combat current and emerging pandemics. We propose strategies to mitigate the negative impact of this ruling on diversifying the infectious disease (ID) workforce.

## Teaser

In this viewpoint, we discuss the consequences of the SCOTUS ruling ending race-conscious consideration for college admission, on diversifying the infectious disease workforce and combatting current and future pandemics. We provide strategies to mitigate this potential negative impact.

## BACKGROUND

Structural racism [1] is deeply embedded in U.S. institutions, including the educational and healthcare systems. In 2003, the Institute of Medicine published the “unequal treatment” report [2] showing that racism in the U.S. healthcare system was a major contributor of racial and ethnic disparities in health outcomes. Twenty years later, the United States has made little progress in closing the racial disparity gap: racial and ethnic minorities continue to experience higher morbidity and mortality from preventable infectious diseases [3]. In 2020, the mortality rate from hepatitis C was twice as high among Black compared with White individuals [4]. Black infants and children have higher odds of death from sepsis compared with their White counterparts [5, 6] and are twice as likely to be hospitalized, require intensive care, and die from influenza-related outcomes [7]. The rate of congenital syphilis is nearly 50 times higher among Black compared with White infants [8]. Furthermore, Black and Hispanic individuals experience disproportionately higher rates of severe acute respiratory syndrome coronavirus 2 infection and coronavirus disease 2019 (COVID-19)-related mortality and exposure risk [9]. In a country that prides itself in advanced scientific discoveries, these persistent disparities underscore how laws and policies create systemically discriminatory conditions for minoritized people. This results in inequitable delivery of healthcare services, including differences in the quality of clinical care received when access is available [10–13]. The challenges and barriers to high-quality care experienced by minoritized people demonstrates the urgent need for an inclusive healthcare system that prioritizes health equity. Furthermore, inequities in infectious diseases outcomes are occurring amid current and projected workforce shortages in ID and poor workforce diversity, which may in turn contribute to continued disparate outcomes in population health [14].

The cost of healthcare is rapidly rising. Underresourced communities with limited access to primary care contribute to a major part of healthcare expenditures [15]. The lack of timely preventive care, poorly managed chronic conditions, late-stage diagnoses, and receipt of poor-quality care among Medicaid recipients are additional factors driving cost among minoritized groups in addition to adverse health outcomes [16, 17]. Inequity in healthcare negatively impacts costs and should be an economic concern for all Americans who directly or indirectly pay higher health care costs.

## STATEMENT OF THE PROBLEM

On 29 June 2023, the U.S. Supreme Court ruled that the race-conscious undergraduate admission programs of Harvard College (6–3) and the University of North Carolina (6–2) violated the Equal Protection Clause of the 14th Amendment [18]. The majority opinion allows for consideration of race holistically within the context of an applicant's individual story. This approach to race-neutrality dismisses the nefarious history [19] and ongoing systemic racist and discriminatory practices across U.S. institutions [2]. By negating the concept of equity (ie, allocation of resources based on need), this ruling provides a blueprint for the dismantling of underrepresented minority in medicine (URiM)-based enrichment programs that have been the main source of the limited diverse healthcare workforce we currently have. By limiting opportunities for higher education for URiM at the undergraduate level, the number of URiM students entering medicine and affiliated fields that feed into the ID workforce (eg, nursing, pharmacy, research, public health) will only decrease. Furthermore, research shows that the disparity in compensation in ID compared with other medical subspecialities contributes to the declining number of trainees entering ID [14, 20]. This is particularly important for URiM students who have less financial and social capital compared with majority students and, on average, graduate with more educational debt [21, 22]. Workforce diversity in healthcare is essential because it plays an important role in reducing health disparities while increasing organizational financial performance, scientific innovation, and impact [23, 24].

## IMPLICATIONS

Increasing the ID workforce diversity requires efforts to increase diversity at all levels of education and training. To understand the potential impact of the Supreme Court of the United States (SCOTUS) ruling on the future ID workforce, it is important to learn from precedent cases. The data are clear: states that dismantled race-conscious admission saw the number of minoritized students accepted at colleges, universities, and medical schools plummet. In the era of Regents of the University of California v. Bakke (1978) [25], the “numbers and proportions of Black, Hispanic, and Native American or Alaska Native (AIAN) medical school matriculants increased, but at a rate slower than their age-matched counterparts in the U.S. population, resulting in increased underrepresentation” [26]. There has been nearly a 44% drop in minoritized student admission in public medical schools in California, Louisiana, Mississippi, and Texas after higher education institutions removed the use of race in admissions decisions [27]. Similarly, following affirmative action bans across 6 states (California, Washington, Florida, Texas, Michigan, and Nebraska), first-time matriculation of URiM medical students decreased by approximately 17.2% [28]. Recent trends are disconcerting: a 2023 study shows that the medical workforce is increasingly composed of students from high-income households, whereas students from low-income families are decreasing by the same rate [29]. These trends are likely to accelerate as the dismantling of race-conscious admission policies are no longer limited to public institutions and in specific states, but now include public and private institutions across the United States.

The United States will face a shortage of ID physicians because of the decreasing numbers of trainees pursuing fellowship training. Match data for 2023 revealed that only 73% and 52% of adult and pediatric ID positions, respectively, were filled [30]. The field of ID is further disproportionately impacted by low URiM enrollment [31]. In 2023, 64 of 472 (13.5%) applicants to adult ID fellowship and 7 of 64 (10.9%) applicants to pediatric ID fellowship were URiM [32, 33]. A decline in the numbers of URiM students matriculating in medical school will result in a decreased pool of potential URiM trainees for the ID workforce. The consequences of fewer ID practitioners and a less diverse ID workforce will negatively impact the national preparedness response to future pandemics. It will decrease our capabilities to control emerging infections resulting from antimicrobial-resistant pathogens. Moreover, the changing patterns in ID brought on by climate change have already increased risks for domestic acquisition of life-threatening infections, such as malaria, which previously were confined to tropical regions.

The immeasurable contribution of ID physicians was clearly demonstrated during the COVID-19 pandemic during which ID clinicians and researchers led multifaceted efforts of the pandemic response, including the development of vaccines that not only saved millions of lives, but importantly reduced hospitalizations and overall healthcare expenditures [34, 35]. Despite the absence of ID physicians in 80% of U.S. counties [36], this underrepresented contingent of the ID workforce led the recruitment of diverse participants in COVID-19 vaccine clinical trials [37–39], and the vaccine uptake increase among minoritized communities [40, 41].

Furthermore, patient–physician racial concordance is associated with improved healthcare use, lower healthcare expenditures in minoritized populations [42], and lower morbidity and mortality [43]. Cultural competency and humility training are necessary interventions [44] but are insufficient to compensate for the value of racial–ethnic physician–patient concordance. Shared life experiences result in a deeper understanding of patient social and cultural norms, more effective bidirectional communication, and higher trust in provider recommendations. Cultural competency and humility can be acquired through training but for this to be done effectively, diversity across all ranks in medicine is required to ensure inclusive voices and perspectives in training, delivery of care, and the development of healthcare policies [45].

## RECOMMENDATIONS

Because the decision to limit race-based consideration in college admissions is our current paradigm, strategies to move forward while continuing to ensure opportunities for URiM students are imperative. The U.S. military has successfully argued that keeping a diverse and inclusive workforce in the military is a matter of national security [18]. We also argue that establishing a diverse, inclusive, and equity-minded healthcare system is a matter of national security. A robust healthcare infrastructure is needed to combat pandemics and other biologic threats. Now is the time to expand, not stifle diversity, equity, and inclusion (DEI) efforts and invest in enrichment, bridge, and pathway programs to recruit and retain URiM trainees and faculty (Table 1). Role modeling and providing learners with the opportunity to be exposed to physicians who look like them and have similar lived experiences is particularly important for URiM learners. However, given the low numbers of URiM ID physicians, it is important that all faculty be involved to alleviate the burden of the “minority tax” on URiM faculty [46]. Student debt is also a factor in specialty selection, underscoring the need to address compensation disparities in ID [21, 22].

**Table 1.** Proposed Strategies to Diversify the Infectious Diseases Workforce Despite Policies Ending Race-Conscious College Admission

Individual Level (Recommendations for ID Practitioners: Physicians, Nurses, Pharmacists)
Role modeling for students and trainees Receive training on how to mentor diverse learners Participate in DEI efforts to create supportive environments for minoritized patients and learners Participate in enrichment and pathway programs for minoritized students
Institutional level (recommendations for ID divisions, hospitals, and medical schools)
Education and training Develop rubrics for holistic review of applicants and provide training for admission/selection committee members Provide universal implicit bias training at all levels along the medical education continuum Develop strategies to recruit and support URiM trainees and faculty Pair with mentorship training of all faculty Develop enrichment programs that target minoritized and underrepresented learners Partner with institutions with high numbers of URiM students Specialty-interest panels and presentations Experiential opportunities (eg, shadowing in ID clinic) Mentored research opportunities Clinical care Develop metrics to measure outcomes of minoritized and underserved patients Implement quality improvement to address disparities in healthcare access and outcomes Assess the use of race in clinical prediction models, algorithms, and screening metrics that may exacerbate health disparities Implement universal assessment of social determinants health during clinical encounters Provide tools and resources to address identified risk factors (eg, access to transportation to appointments)
National level (recommendations for ID professional organizations)
Provide conference scholarships to underrepresented learners Provide mentorship programs to underrepresented learners and faculty Develop collaborations with underrepresented physician and medical student organizations Continue advocacy for equitable compensation

Abbreviations: DEI, Diversity, Equity, and Inclusion; ID, infectious diseases; URiM, underrepresented minority in medicine.

The SCOTUS ruling allows for holistic consideration of race within the context of an applicant's individual story. Admissions committees should, therefore, adopt a structured mission-based “holistic review” review process to mitigate the subjectivity biases of an unstructured approach [47]. It will be important to develop rubrics for holistic review of applicants across diverse educational levels (from the undergraduate to graduate) and provide training for selection committees. We must increase the pool of diverse applicants by expanding academic enrichment programs with structured longitudinal mentoring and coaching that address the social, environmental, and financial determinants of academic success [48]. We must build programs that consider conditions rooted in structural racism that ultimately disproportionately impact minoritized groups; low socioeconomic status, limited educational opportunities, poor housing conditions, overpolicing, English language proficiency, and immigration status. Collaboration with institutions with high numbers of URiM students could help increase exposure to and inspire interest in the field of ID [49]. This approach has been used with Historically Black Colleges and Universities to provide exposure to emergency medicine [50]. Such collaborations must be paired with creating a culture of inclusivity within institutions of learning, a vital part of increasing and maintaining ID physician workforce diversity [51].

On a national organization level, URiM scholarships to attend ID conferences can be offered and should be paired with mentorship workshops. The “meetID” program by the Pediatric Infectious Diseases Society is one such example [52]. The Infectious Diseases Society of America has the Grants for Emerging Researchers/Clinicians Mentoring (G.E.R.M.) program that supports a longitudinal ID-mentored research project; such programs can be offered by other organizations and specifically support URiM learners [53]. National organizations should also develop partnerships with URiM physician and medical student organizations, such as the National Medical Association, National Hispanic Medical Association, Student National Medical Association, and the Latino Medical Student Association, to name a few. Such organizational collaborations can be used to develop experiential and mentored research opportunities in ID for URiM students and trainees.

## CONCLUSION

Infectious agents do not discriminate based on race or ethnicity. The disparities in ID that the United States experiences directly reflect the inequities in access and receipt of preventive and curative treatments. Healthcare disparities have profound health and cost implications for all and having a robust and diverse healthcare workforce positions our society to combat the drivers of inequity from a place of strength. Given the multiple a priori barriers faced by aspiring URiM physicians desiring ID specialization, we cannot choose to be passive and watch the dismantling of DEI efforts that will ultimately contribute to poor health outcomes for minoritized groups and higher healthcare costs. We must be proactive, intentional, and committed in our resolve to advance health equity in light of the SCOTUS ruling.

As Helene D. Gayle, MD, MPH, first director of the Centers for Disease Control and Prevention National Center on HIV, TB and STD Prevention and current president of Spelman College, states: “It is critical that we do not allow the recent SCOTUS decision to impede the important efforts to address the glaring health disparities that exist in our country and the critical need for having a more diverse health workforce.”
